# Quercitrin, the primary active ingredient of *Albizia julibrissin* Durazz. flowers, alleviates methamphetamine-induced hepatotoxicity through a mitochondria-mediated apoptosis pathway

**DOI:** 10.3389/fphar.2025.1482172

**Published:** 2025-03-03

**Authors:** Dan Wu, Bo Xie, Jing Li, Zhangang Xiao, Jing Shen, Xu Wu, Mingxing Li, Qin Sun, Hongping Shen, Xiaobing Li, Yong Dai, Yueshui Zhao

**Affiliations:** ^1^ Integrated Chinese and Western Medicine School, Southwest Medical University, Luzhou, China; ^2^ Laboratory of Molecular Pharmacology, Department of Pharmacology, School of Pharmacy, Southwest Medical University, Luzhou, China; ^3^ Cell Therapy and Cell Drugs of Luzhou Key Laboratory, Southwest Medical University, Luzhou, China; ^4^ South Sichuan Institute of Translational Medicine, Luzhou, China; ^5^ Department of Oncology and Hematology, The Affiliated Traditional Chinese Medicine Hospital, Southwest Medical University, Sichuan, China; ^6^ National Traditional Chinese Medicine Clinical Research Base, Affiliated Traditional Chinese Medicine Hospital, Southwest Medical University, Luzhou, China; ^7^ Drug Research Center of Integrated Traditional Chinese and Western Medicine, Affiliated Traditional Chinese Medicine Hospital, Southwest Medical University, Luzhou, China; ^8^ Sichuan Police College, Luzhou, Sichuan, China

**Keywords:** quercitrin, oxidative damage, hepatotoxicity, ROS, apoptosis

## Abstract

**Background and purpose:**

Methamphetamine (METH), a synthetic psychostimulant and highly addictive drug, could cause depression and acute liver injury. There have been few studies on the mechanism by which METH induces liver damage and on how to alleviate METH-induced hepatic toxicities. *Albizzia julibrissin* Durazz. flowers (AF) is a traditional Chinese medicine known for its ability to releve depression and soothe the liver. The extracts of AF have shown hepatoprotective effects with their anti-oxidative activities. The potential of AF extracts to alleviate METH-induced hepatic toxicity remains unclear. This study aims to investigate the effects of AF extracts and their priamry active ingredient on METH-induced hepatotoxicity and explore the potential underlying mechanisms.

**Methods:**

Firstly, we used the MTT assay to screen the active components of AF. Then, UPLC-MS/MS was employed to analyze the effective components and identify their activities. In addition, *in vitro* and *in vivo* experiments were conducted to explore the effects of the active components on METH-induced hepatic toxicity. Moreover, flow cytometry was employed to detect the effects of the active components of AF on METH-induced hepatocyte cycle arrest and apoptosis; biochemical kits were used to detect oxidative damage; transmission electron microscopy, mitochondrial membrane potential probes, and Western blotting were used to analyze mitochondrial damage. C57/BL6J mice were used to establish a METH-mediated acute liver injury model. After 21 days of intervention with the effective components of AF, serum from mice was collected to detect the level of liver injury markers, and tissues were collected for H&E staining, oxidation index analysis, and mitochondrial-related protein expression analysis.

**Results:**

We found that the ethyl acetate fraction of *AF* extracts significantly alleviated the decrase in hepatocyte activity induced by METH *in vitro*. Further UPLC-MS/MS analyses showed that quercitrin (QR) is the major active ingredient of *AF extracts*. QR alleviates METH-induced hepatocyte apoptosis, cell cycle arrest, oxidative stress, and mitochondrial damage. QR alleviates METH-induced oxidative liver damage in mice and exerts therapeutic effects by regulating the BAX/CASP3 pathway.

**Conclusion:**

*AF* and its main component QR can effectively alleviate METH-induced liver injury, and its mechanism is related to the mitochondria-mediated apoptotic pathway.

## 1 Introduction

Methamphetamine (METH), commonly known as “crystal meth,” is a synthetically manufactured psychostimulant drug. It has dominated both domestic and international drug markets in recent years (Hong et al., 2021). Short-term, high-dose METH intake can cause damage to the nervous system and other organs throughout the body (Prakash et al., 2017; Yang et al., 2018). The liver, serving as the primary detoxification and metabolic organ, is one of the first organs to be affected (Volkow et al., 2010).

METH abuse leads inevitably to acute hepatic injury. In severe cases, it can result in acute liver failure and even mortality (Volkow et al., 2010; Eskandari et al., 2014). The mechanism of METH-induced hepatic injury is closely associated with oxidative stress and mitochondrial damage (Koriem and Soliman, 2014; Mashayekhi et al., 2014). Currently, effective treatments for drug-induced liver injury of this nature are lacking.

The Chinese medicine *Albizia julibrissin* Durazz. flowers (AF) is the dried inflorescences or flower buds of the leguminous plant *Albizia julibrissin* Durazz.*,* which is widely used in traditional medicine to treat a wide range of ailments, including depression, insomnia, fever, abscesses, pain, and rheumatism (Tang W, 2011; Chang et al., 2019; Huang et al., 2023). Various bioactive components of AF have been isolated and identified, such as triterpenoid saponins, flavonoids, lignans, alkaloids, and glycosides. (Zheng et al., 2006; He et al., 2020; Lu et al., 2023).

Several studies have highlighted the protective effects of *Albizia julibrissin* flowers, particularly their antioxidant, anti-inflammatory, and neuroprotective properties. The antioxidant activity is attributed to the high flavonoid content, which can neutralize free radicals and reduce oxidative stress, potentially preventing cellular damage like apoptosis in various tissues (Harihar et al., 2024). Additionally, AF extracts showed anti-inflammatory effects by inhibiting the generation of pro-inflammatory cytokines and enzymes, which may help in managing inflammatory diseases like arthritis (Harihar et al., 2024). Furthermore, AF extracts have shown promise in neuroprotection, reducing neuroinflammation and oxidative damage, which may offer therapeutic potential in neurodegenerative diseases, such as Alzheimer’s disease (Harihar et al., 2024). These protective effects are likely mediated through the modulation of cellular signaling pathways, including those involving Nrf2, which is known for regulating the expression of antioxidant genes (Cheng et al., 2024).

The purpose of this study is to investigate the effects of AF extracts, especially the effects of their major bioactive component(s), on METH-induced liver injury and the underlying mechanisms. Findings from this study may provide a theoretical basis for the development of new drugs to treat METH-induced liver damage.

## 2 Materials and methods

### 2.1 Chemicals and reagents

*Albizia julibrissin* Durazz. flowers were from Sichuan Tianshui Traditional Chinese Medicine Co., Ltd (Sichuan, China) (article number:22100101). The heavy metals, microorganisms, insecticides and other contaminants of the drug were detected. All test results were in line with the regulations formulated by the Chinese government. Quercitrin reference substance was from Chengdu Purifa Technology Development Co., Ltd (Sichuan, China). METH is provided by Sichuan Police College. Kits for assays of aspartate aminotransferase (AST), alanine aminotransferase (ALT), superoxide dismutase (SOD), glutathione (GSH), and malondialdehyde (MDA) were from Nanjing Jiancheng Bioengineering Institute (Nanjing, China). Rh123 fluorescent dye and thiazole blue were from Shanghai Macklin Biochemical Technology Co., Ltd. (Shanghai, China). The ANNEXIN V-FITC/PI apoptosis detection kit was from Beijing Solarbio Science & Technology Co., Ltd. (Beijing, China). Hoechst 33342 nuclear dye was from 4A Biotech Co., Ltd. (Suzhou, China). Mito-Tracker Red CMXRos (mitochondrial red fluorescent probe), BCA protein quantitative kit, HPR labeled goat anti-rabbit IgG antibody and HPR labeled goat anti-mouse IgG antibody were from Beyotime Biotechnology (Shanghai, China). Antibodies for Bax, Bcl-2, Caspase-3, and GAPDH were from Cell Signaling Technology Inc (Denver, Massachusetts, United States). ECL chemiluminescence kit was from Bio-Rad Laboratories, Inc (Hercules, California, United States). All HPLC grade solvents were purchased from Thermo Fisher Scientific Inc (Waltham, Massachusetts, United States).

### 2.2 Separation of quercitrin (QR) from AF

The isolation method for the major component of AF was adapted from Xiong et al. (2021). In brief, 2 kg of dried AF were moderately crushed and soaked in a 70%–80% ethanol solution for 3–4 days. The soaking solution was collected and filtered using defatted cotton. Then a rotary evaporator was used for decompression and recovery, and the concentrated solution was collected. The remaining medicinal materials were treated 3∼4 times with the same method, and the concentrated solutions were combined. The combined concentrated solution was subsequently extracted with petroleum ether, ethyl acetate, n-butanol, and each extract was recovered under reduced pressure. Then, each part of the concentrate was freeze-dried to obtain each part of the extract. The ethyl acetate extract was further separated on a silica gel column (100–200 mesh), and three fractions were obtained by gradient elution with chloroform-methanol (20:1∼2:1). The solvent of fraction 2 was recovered, and the remaining extract was fully mixed with chloroform-methanol (1:1) solution and filtered after standing overnight. The filtrate residue was dried and obtained a yellow powder. The purity was detected by 1260 high performance liquid chromatograph (HPLC) (Agilent, California, United States), and the structure was analyzed by ultra-high performance liquid chromatography tandem high resolution mass spectrometer.

### 2.3 Cell culture and cell viability detection

The human normal liver cell lines LO-2 and MIHA cells (iCell Bioscience Inc., Shanghai) were cultured in an incubator maintained at 37°C with 5% CO_2_, with DMEM and RPMI-1640 medium supplemented with serum and two antibiotics (penicillin-streptomycin solution).

The viability of LO-2 and MIHA cells affected by *AF* alcohol extract (AFAE), the ethyl acetate (EA) fraction of the AF extract, QR, and METH was evaluated via the MTT assay. Cells were seeded in 96-well plates at densities of 2.5 × 10^3^/well, and 4 × 10^3^ cells/well, respectively. The cells were divided into control, model (METH), and treatment groups.

In addition to the control group, METH (2.5 mM) was added to each group. After 24 h of incubation, cells in the treatment group were exposed to different concentrations of AFAE, EA fraction of AF extract, and QR (10, 50, 100, 200 μg/mL). Following an additional 24 h of incubation, MTT solution (5 mg/mL) was added to each well and incubated for 4 h. Absorbance at 490 nm was then measured using a microplate reader.

### 2.4 Apoptosis and cell cycle analyses

LO-2 and MIHA cells were cultured in six-well plates and divided into four groups: control, model (METH), treatment (METH + QR), and QR alone. METH (2.5 mM) was added to each well except for the control group. After 24 h of incubation, cells in the treatment and QR alone groups were treated with QR (200 μg/mL) for an additional 24 h. Subsequently, cells were collected and washed twice with phosphate-buffered saline (PBS) (pH 7.4). Cell apoptosis was assessed using an Annexin V-FITC/PI apoptosis detection kit following the manufacturer’s instructions and analyzed by flow cytometry.

The method for collecting cells for the cell cycle experiment was identical to that used for the apoptosis experiment. Cells were labeled with propidium iodide (PI) staining solution following the manufacturer’s instructions. Flow cytometry was employed to measure the DNA content of cells in the G_0_/G_1_, S, and G_2_/M phases. Subsequently, Flowjo software was used to analyze the results of both cell apoptosis and cell cycle.

### 2.5 Measurement of ROS level and mitochondrial membrane potential analyses

#### 2.5.1 Analysis of ROS levels

In live cells, dichlorodihydrofluorescein diacetate (DCFH-DA) is cleaved by esterases to form dichlorodihydrofluorescein (DCFH), which is then oxidized by ROS to generate the highly fluorescent molecule 2′,7′-dichlorofluorescein (DCF) (Furger, 2021). The DCFH-DA method was selected to detect intracellular ROS levels. LO-2 and MIHA cells were seeded into 96-well plates at densities of 2.5 × 10^3^ and 4 × 10^3^ cells/well, respectively, and divided into control, model (METH), treatment (METH + QR), and QR groups.

In addition to the control group, METH (2.5 mM) was added to each well. After 24 h of incubation, cells in the treatment and QR groups were treated with QR (200 μg/mL) for 24 h. Subsequently, a ROS solution (10 μM) was added to each well and incubated with the cells for 15 min. Following the removal of supernatant, cells were washed twice with PBS, and fresh culture medium was added. The fluorescence of the cells was observed using a fluorescence microscope (Nikon Instruments, Tokyo, Japan) with specific laser excitation, and the average fluorescence intensity was calculated using ImageJ software.

#### 2.5.2 Measurement of mitochondrial membrane potential

Mitochondrial membrane potential detection: Cell preparation was conducted following the same procedure as for ROS detection. After incubation, Rhodamine 123 (Rh123) or MitoTracker Red CMXRos (5 μM) detection solution was added to each well and incubated in the dark for 30 min. Subsequently, cells were washed twice with PBS, and Hoechst 33342 dye (5 μM) was added for incubation in the dark for an additional 15 min. After washing twice with PBS, fresh culture medium was added. Fluorescence was excited and imaged under a fluorescence microscope.

### 2.6 Observation of hepatocyte mitochondria by transmission electron microscope (TEM)

After grouping and culturing LO-2 cells, the culture medium was discarded, and they were treated with electron microscopy fixative. The cells were gently scraped off and collected, centrifuged at 1,000 rpm for 5 min, fixed with glutaraldehyde, and then fixed in 2% osmium tetroxide (O_S_O4). After dehydration with a graded alcohol series, the cells were embedded in resin, and ultrathin sections were obtained. The sections were stained with uranyl acetate and lead citrate. Changes in mitochondrial morphology were observed under a transmission electron microscope (JEM-1230, Tokyo, Japan).

### 2.7 Animals experiments

Male C57BL/6J mice (SPF grade, 6∼8 weeks old & 17∼21 g) were from SPF (Beijing) Biotechnology Co., Ltd. Animals were fed in the Experimental Animal Center of Southwest Medical University, with *ad libitum* access to food and water. Room conditions were maintained at a temperature of 21∼25°C and relative humidity of 55%∼60%, with a 12-h light-dark cycle. Following a one-week adaptation feeding, the subsequent experiments were conducted. All experimental procedures were approved by the Ethics Committee for Animal Research of Southwest Medical University (Approval No.: 20220930-026).

After one week of adaptive feeding, 28 mice were randomly divided into 4 groups (n = 7): control group, model group (20 mg/kg METH group), low-dose treatment group (METH + 100 mg/kg QR) and high-dose treatment group (METH + 200 mg/kg QR).

METH (20 mg/kg) was dissolved in sterile saline. Two concentrations of QR were prepared into a suspension containing 0.5% (w/v) CMC-Na. METH (20 mg/kg, dissolved in sterile saline) was intraperitoneally injected once daily for 15 consecutive days. Following the 15-day METH treatment period, the QR treatment groups were intragastrically administered with QR (100 mg/kg or 200 mg/kg, suspended in 0.5% (w/v) CMC - Na) for 21 days. During this 21-day period, the control group and the METH group were treated with normal saline.

All mice were sacrificed within 6∼12 h after the end of the treatment group, and their blood samples and liver tissues were collected for further analysis. Blood samples were centrifuged (4°C, 3,500 rpm, 10 min) to separate serum and stored at −20°C. After weighing the liver tissue, one part was fixed in 4% paraformaldehyde and the other part was stored at −80°C.

### 2.8 Hematoxylin and eosin (H&E) staining

The liver tissues were fixed with 4% paraformaldehyde for 24 h and then embedded in paraffin. Next, the tissues were cut into 5 μm sections. H&E staining was performed and photographed under the microscope.

### 2.9 Serum ALT and AST measurements

Serum alanine aminotransferase (ALT) and aspartate aminotransferase (AST) were measured according to the manufacturer’s instructions using a biochemical kit (Nanjing Construction Bioengineering Institute, China).

### 2.10 Determination of SOD, GSH and MDA levels in liver tissue

An appropriate amount of thawed frozen liver tissue was taken, homogenized with 9 times the volume of 4°C saline and centrifuged (4°C, 3,500 rpm, 10 min). After centrifugation, supernatants were collected for further analyses. The protein concentration of the liver homogenate was measured using a BCA protein concentration assay kit. SOD, GSH, and MDA levels of liver homogenates were determined according to the instructions of the related kits (Nanjing Construction Bioengineering Institute, China).

### 2.11 Western blot analysis

Total protein extracts of the cells were prepared using RIPA lysis buffer. Protein concentrations in all samples were determined using the BCA Protein Concentration Assay Kit. A small amount of total protein (30 μg) from each sample was subjected to sodium dodecyl sulfate-polyacrylamide gel electrophoresis (SDS-PAGE) and electrophoretically transferred to a polyvinylidene fluoride (PVDF) membrane (Immobilon®-P, United States). The membranes were incubated with 5% (w/v) skimmed milk in PBS buffer containing Tween-20 (1‰) for 90 min at room temperature and with different primary antibodies at 4°C. The primary antibodies of anti-Bax, anti-Bcl-2, anti-Caspase-3, anti-GAPDH were used at concentrations of 1:1000. The membranes were then washed three times and incubated with goat anti-rabbit IgG-HRP secondary antibody (1:5,000) for 50 min at room temperature. GAPDH was used as an internal reference protein for total protein blotting. The developer solution was prepared with ECL and ECL+ (1:1) and incubated with the membrane for 30 s∼90 s in a developer (ChemiScope 6100, Shanghai, China). ImageJ software was used to quantify the density of the bands.

### 2.12 Statistical analysis

The experimental data were analyzed using GraphPad Prism 9 software, and the results were presented as “mean ± standard deviation (SD).” Differences between two groups were compared using the t-test, while differences among multiple groups were assessed using one-way or two-way analysis of variance (ANOVA). A p-value less than 0.05 was considered statistically significant.

## 3 Results

### 3.1 The active ingredient of AF, quercitrin, alleviated METH-induced hepatotoxicity

In order to understand the effects of different concentrations of METH and AFAE on the viability of LO-2 cells and MIHA cells, MTT assay was used to detect the effects of different concentrations of METH (0, 0.5,1, 2, 3, 4, 5, 6, 7 mM) on the viability of LO-2 cells and MIHA cells.

**FIGURE 1:**
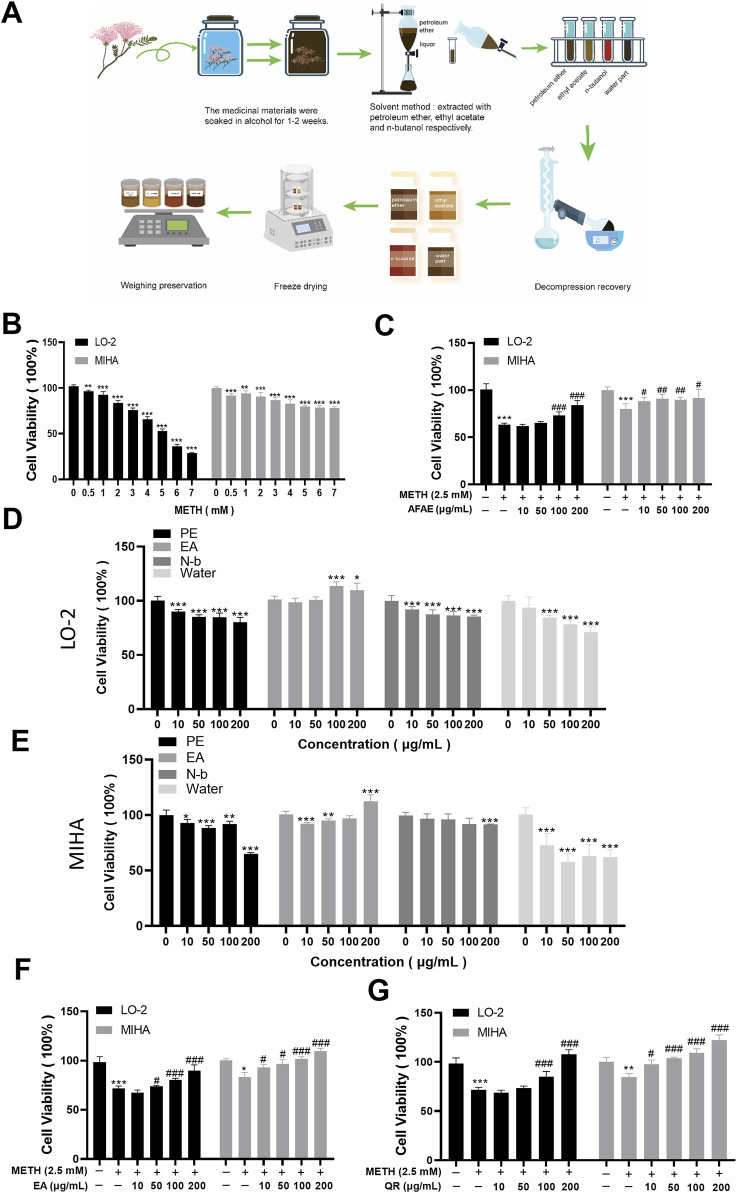
AF extraction and its active component, QR, alleviate METH-induced hepatic cell viability decrease. **(A)** Flowchart of process of *AF* active component extraction. **(B)** Effects of METH with different concentrations on hepatocyte proliferation. **(C)** Effects of AF alcohol extract on METH-induced hepatocyte damage. **(D, E)** Effects of AF different fractions, including petroleum ether (PE), ethyl acetate (EA), n-butanol (N-b), and aqueous extract, on hepatocytes. **(F)** Effects of EA extract of AF on METH-induced hepatocyte damage. **(G)** Effects of QR on METH-induced hepatocyte damage. Data were shown as mean ± SD of three independent experiments. *P < 0.05, **P < 0.01, ***P < 0.001, compared with control group. ^#^P < 0.05, ^##^P < 0.01, ^###^P < 0.001, compared with METH-treated group.

The results, as shown in Figure 1B (**P < 0.01, ***P < 0.001,compared with control group), showed that after 24 h, the cell viability in the METH group decreased in a concentration-dependent manner. The cell viability of LO-2 cells was 60%∼70%, while the cell viability of MIHA cells was 70%∼80%, when cells were treated with 2.5 mM of METH. Therefore, we used 2.5 mM of METH for subsequent *in vitro* studies.

The effects of AFAE on METH-induced hepatocyte injury were investigated (Figure 1C). Hepatocyte viability in the METH group was significantly reduced. After intervention with different concentrations of AFAE, hepatocyte viability increased remarkably in a dose-dependent manner (***P < 0.001, compared with control group, #P < 0.05, ##P < 0.01, ###P < 0.001, compared with METH-treated group). These experimental findings suggest that AFAE can mitigate METH-induced hepatocyte viability damage. To further investigate the primary active constituents of AF, the extract was fractionated into four components: petroleum ether (PE), ethyl acetate (EA), n-butanol (N-b), and the water part. And the extraction procedure was illustrated in Figure 1A.

To identify effective components, our subsequent step involved an examination of the four fractions’ effects on the viability of normal hepatic cells (Figures 1D, E). Within the range of 10∼200 μg/mL, the PE, N-b, and the water part of AFAE showed notable reduction in hepatic cell activity, indicating cytotoxic effects (*P < 0.05, **P < 0.01, ***P < 0.001, compared with control group). Conversely, the ethyl acetate fraction at concentrations within the same range stimulated hepatic cell activity without evident cytotoxicity. Co-treatment of the ethyl acetate fraction with METH (Figure 1F) led to a notable enhancement in hepatic cell activity when compared to the METH group, demonstrating a dose-dependent increase with the inclusion of different concentrations of the ethyl acetate fraction (*P < 0.05, ***P < 0.001, compared with control group, #P < 0.05, ###P < 0.001, compared with METH-treated group). These findings suggest the therapeutic efficacy of the ethyl acetate fraction of AFAE against METH-induced impairment of hepatic cell viability, underscoring it as the active component of AF.

Next, we analyzed the EA fraction, isolated its primary active ingredient, quercitrin (QR), and identified it.

**FIGURE 2:**
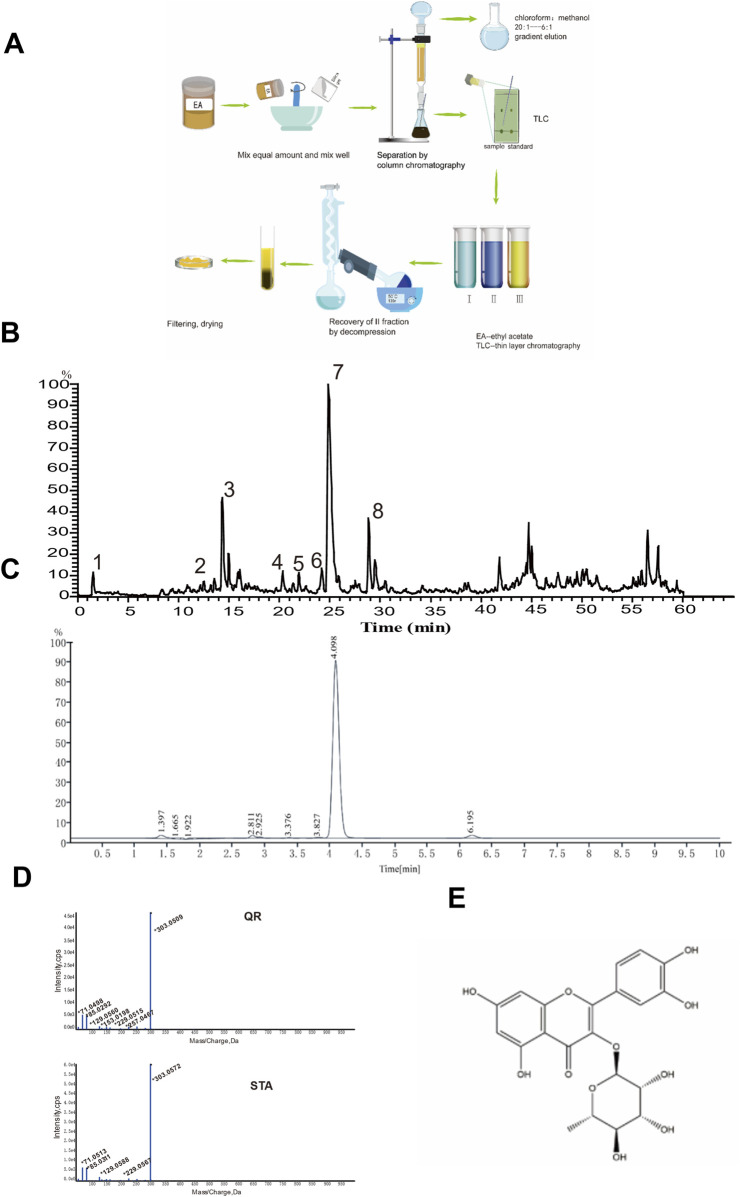
UPLC-MS/MS analysis of the EA fraction of *AF* and the separation/identification of QR. **(A)** Flowchart of QR separation. **(B)** Total ion chromatogram of the EA fraction of AF in positive ion mode. **(C)** Chromatogram of QR with UPLC analysis. **(D)** The secondary mass spectra of QR and of the standard QR sample. **(E)** Structural diagram of QR.

To further understand the composition of the EA fraction of AF, we employed UPLC-MS/MS analyses (Figure 2B). Our analysis, based on database comparisons and existing literature, revealed eight compounds present in the EA fraction, including L-valine, L-phenylalanine, camphor, myricitrin, hyperoside, guaijaverin, quercitrin (QR), and afzelin (Table 1) (Arima and Danno, 2002; Eskandari et al., 2014; Zhu et al., 2017; Wen et al., 2018; Pacheco et al., 2021; Park, 2022; Sayuti et al., 2023). Notably, QR showed the highest peak area, exceeding 55%, prompting further investigation into its properties (Table 1).

**TABLE 1:** List of compounds identified in EA part by UPLC–MS/MS analyses.

No.	RT (min)	Compound name	Error (ppm)	Molecular fomula	Peak area (%)	References
1	1.56	L-Valine	0.8	C_5_H_11_NO_2_	0.3	Zhu et al. (2017)
2	13.37	L-Phenylalanine	−2.5	C_9_H_11_NO_2_	0.3	Zhu et al. (2017)
3	14.32	alcanfor	−1.9	C_10_H_16_O	31	Eskandari et al. (2014)
4	20.33	Myricitrin	−3.2	C_21_H_20_O_12_	3	Park SK (2022)
5	21.90	Hyperoside	−3.2	C_21_H_20_O_12_	3	Wen et al. (2018)
6	24.27	Guajavarin	−3.2	C_20_H_18_O_11_	1.4	Arima and Danno (2002)
7	24.91	Quercitrin	−4.2	C_21_H_20_O_11_	55	Pacheco et al. (2021)
8	28.83	Afzelin	−3.0	C_21_H_20_O_10_	6	Sayuti et al. (2023)

QR was subsequently isolated and identified from the EA fraction using column chromatography (Figure 2A). The resulting yellow powder, soluble in methanol, demonstrated approximately 95% purity via high-performance liquid chromatography (HPLC) (Figure 2C). Thin-layer chromatography, when compared with QR reference standards, exhibited identical chromatographic behavior. High-resolution electrospray ionization mass spectrometry (HR-ESI-MS) yielded a m/z of 449.1059 [M + H]^+^ (calculated value 449.1078), indicating a molecular composition of C_21_H_20_O_11_. The chromatographic retention time perfectly matched that of the QR reference standard. Mass spectral data corroborated the identity of QR (Figure 2D), consistent with literature reports (Pacheco et al., 2021). Therefore, the yellow powder was conclusively identified as QR, as illustrated by its structural formula in Figure 2E (Wu et al., 2010).

To assess the efficacy of QR, we evaluated its effects on METH-induced hepatocellular damage by MTT assay (Figure 1G). Compared to the METH group, QR significantly reversed METH-induced decrease of liver cell activity (**P < 0.01, ***P < 0.001, compared with control group, #P < 0.05, ###P < 0.001, compared with METH-treated group).

These findings suggest that QR effectively alleviates METH-induced hepatocellular toxicity.

### 3.2 QR suppressed METH-induced hepatocyte apoptosis and G_0_/G_1_ phase arrest

**FIGURE 3:**
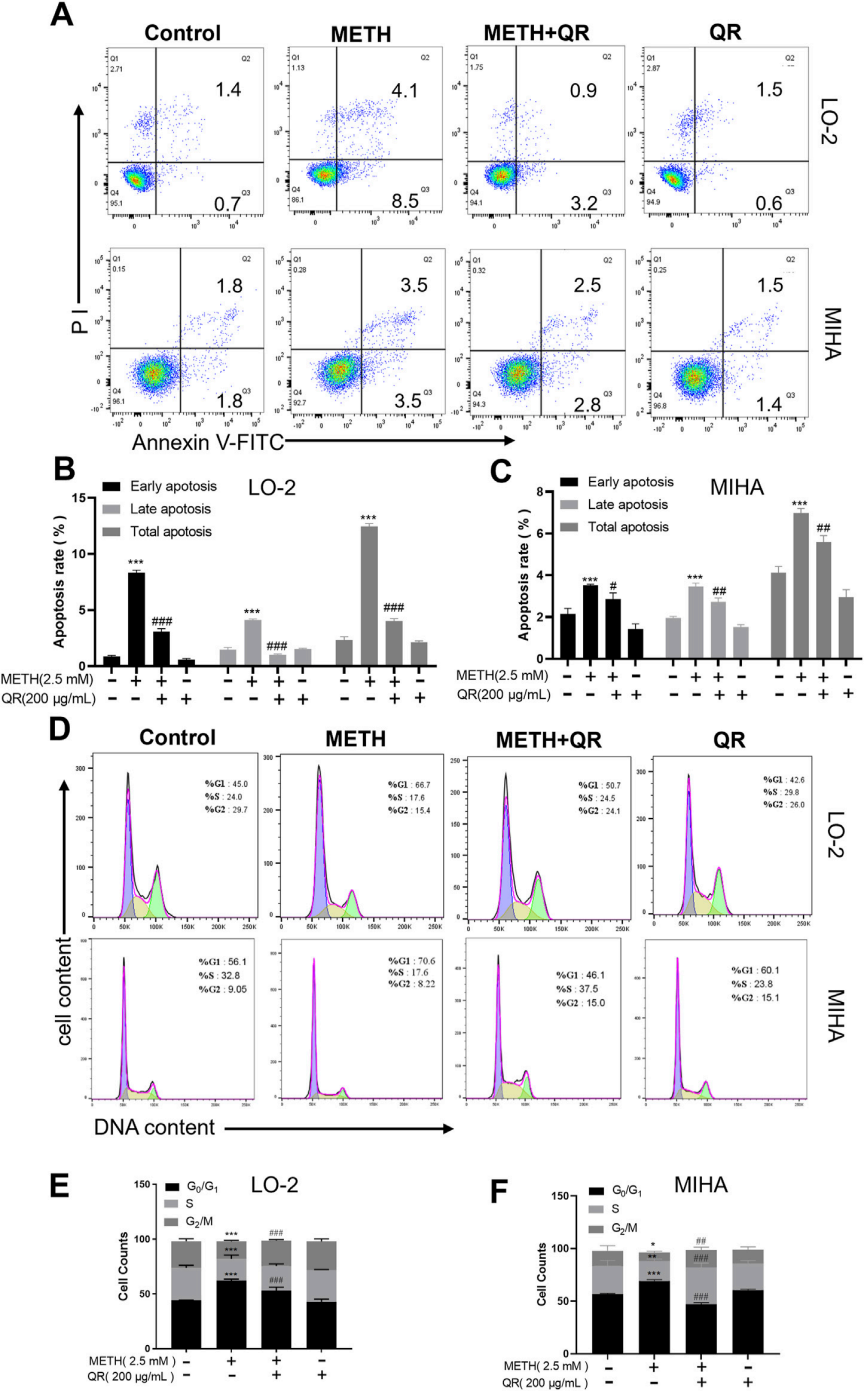
QR alleviates METH-induced apoptosis and G0/G1 phase cell cycle arrest of hepatocytes. **(A)** Apoptosis anlyses of hepatocytes by flow cytometry after METH and QR treatment. **(B, C)** Histograms of early apoptosis, late apoptosis, and total apoptosis analyses of hepatocytes after METH and QR treatment. **(D)** Cell cycle distribution of hepatocytes after METH and QR treatment. **(E, F)** Bar graphs depicting the results of G_0_/G_1_, S, and G_2_/M phases of hepatocytes after METH and QR treatment. Data were shown as the mean ± SD of three independent experiments. *P < 0.05, **P < 0.01, ***P < 0.001, compared to control group. ^##^P < 0.01, ^###^P < 0.001, compared to METH-treated group.

We investigated the effects of QR on METH-induced apoptosis and cell cycle of hepatocytes via flow cytometry analyses. As shown in Figures 3A–C, METH treatment induced the overall apoptotic level of cells, while QR treatment resulted in a decrease in apoptotic levels when compared with the METH group (***P < 0.001, compared with control group, #P < 0.05, ##P < 0.01, ###P < 0.001, compared with METH-treated group).

Additionally, as illustrated in Figures 3D–F, METH treatment of hepatocytes led to a significant increase in the proportion of cells in the G_0_/G_1_ phase of the cell cycle after 24 h, accompanied by a decrease in the number of cells in S phase, indicating G_0_/G_1_ phase cell cycle arrest. However, following 24 h of QR treatment, the proportion of cells in G_0_/G_1_ phase decreased compared to the METH group, along with a decrease in cell numbers in S phase (*P < 0.05, **P < 0.01, ***P < 0.001, compared with control group, ##P < 0.01, ###P < 0.001, compared with METH-treated group), suggesting that QR alleviated METH-induced G_0_/G_1_ phase cell cycle arrest of hepatocytes.

### 3.3 QR alleviated METH-induced oxidative damage and mitochondrial dysfunction of liver cells

**FIGURE 4:**
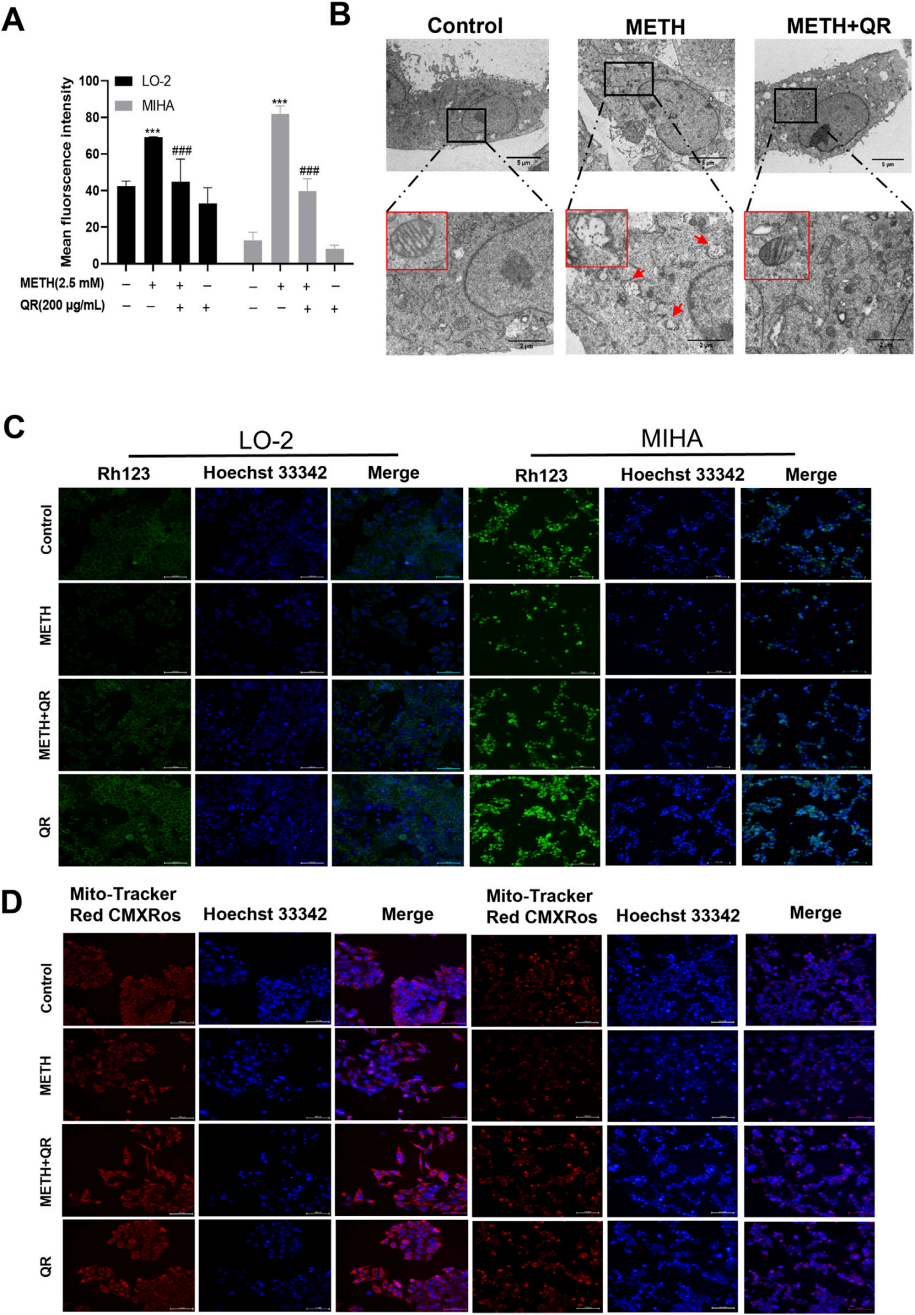
QR ameliorates METH-induced oxidative damage and mitochondrial dysfunction of hepatocytes. **(A)** ROS levels of QR-treated and METH-treated hepatocytes. **(B)** Transmission electron microscopy results of hepatocytes after treatment with QR and/or METH (red arrows indicate ruptured and missing mitochondrial cristae). **(C, D)** Fluorescence microscopy images of hepatocytes treated with METH and/or QR, cells were stained with Rhodamine 123 and Mito-Tracker Red CMXRos (mitochondrial red fluorescence probe) in conjunction with Hoechst 33342. Data were shown as the mean ± SD of three independent experiments. ***P < 0.001, compared to control group. ^###^P < 0.001, compared to the METH-treated group.

To further investigate the effect of QR on METH-induced oxidative damage of liver cells, intracellular ROS levels (indicated by fluorescence intensity) were analyzed. As shown in Figure 4A, compared to control group, the ROS levels of METH-treated group were significantly increased. However, after QR treatment, ROS levels decreased dramatically (***P < 0.001, compared with control group, ###P < 0.001, compared with METH-treated group).

ROS are primarily generated in mitochondria, and previous studies have shown that METH induces mitochondrial damage in liver cells, leading to a decrease in mitochondrial membrane potential (Nakagawa et al., 2009). To investigate the effect of QR on METH-induced mitochondrial damage, liver cell transmission electron microscopy analysis was conducted. Results showed that METH-treated cells exhibited mitochondrial cristae disorganization, rupture, loss, and the presence of mitochondrial vacuoles, indicating mitochondrial damage (Figure 4B). In contrast, cells of QR-treated group showed a reduction in the degree of mitochondrial rupture and vacuolization (Figure 4B). Subsequently, changes in mitochondrial membrane potential of liver cells after METH and QR treatment were assessed using Rhodamine 123 and mitochondrial red fluorescence probes, respectively (Figures 4C, D). The fluorescence intensity of METH-treated group was significantly lower than that of control group, indicating a decrease in mitochondrial membrane potential. However, the fluorescence intensity of QR-treated group was significantly enhanced compared to the METH-treated group, indicating a restoration of mitochondrial membrane potential. These results collectively suggest that QR alleviates METH-induced oxidative stress and mitochondrial damage of liver cells.

### 3.4 QR alleviated the acute liver injury induced by METH in mice

**FIGURE 5:**
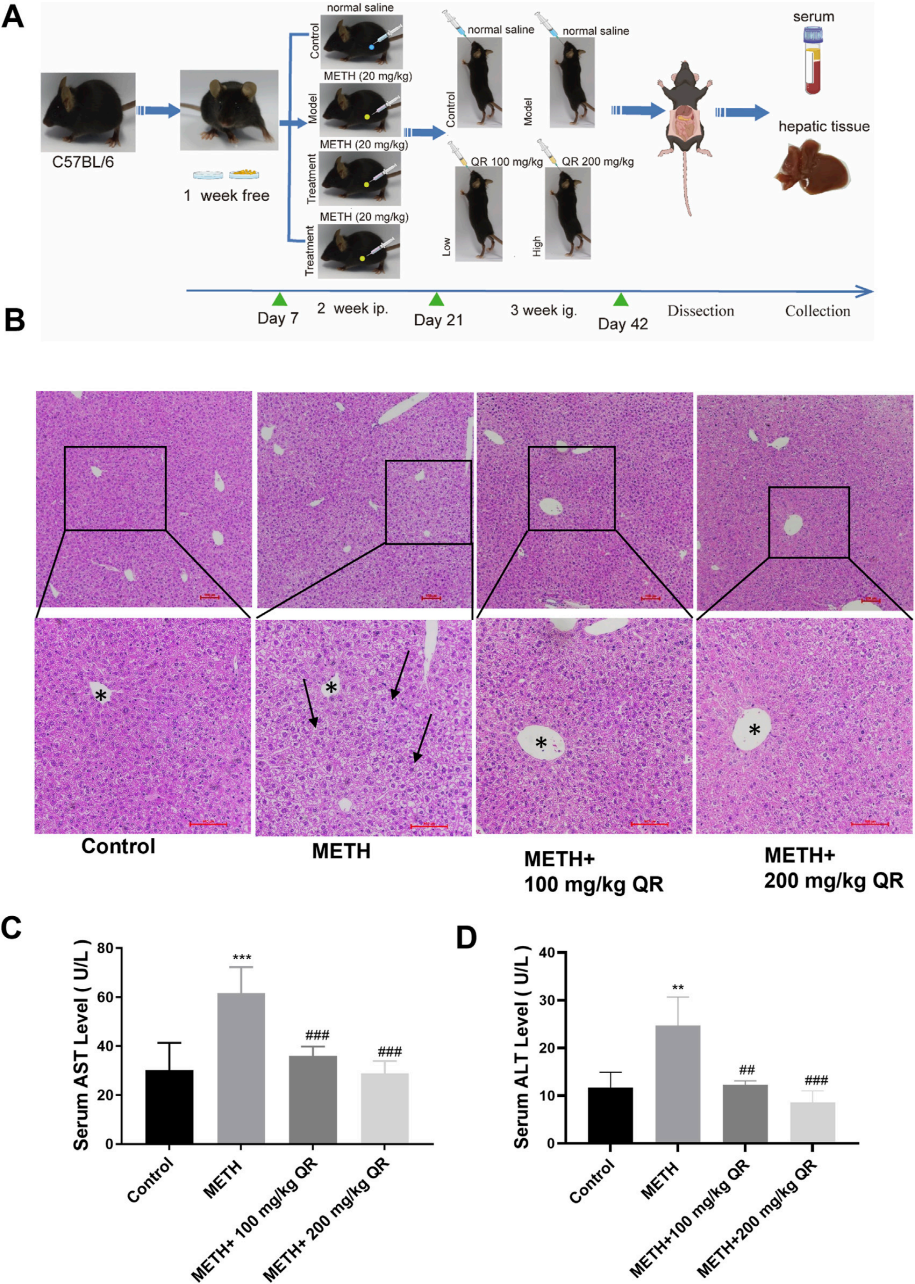
QR alleviates METH-induced acute liver injury in mice. **(A)** Experimental procedure for animal studies. **(B)** H&E staining for mouse liver tissue sections (black arrows indicate extensive cytoplasm disappearance; * indicates central hepatic vein). **(C, D)** Serum levels of ALT and AST. Data were shown as the mean ± SD of three independent experiments. **P < 0.01, ***P < 0.001, compared with control group. ^##^P < 0.01, ^###^P < 0.001, compared with METH-treated group.

Then, we investigated whether QR could alleviate METH-induced acute liver injury *in vivo*. The flowchart of the animal experiment is depicted in Figure 5A. Histopathological examination of mouse liver tissue stained with H&E revealed that, in livers of control group, hepatocytes showed irregular polygonal shapes, with round nuclei located in the center of the cells; meanwhile hepatocytes were arranged in cord-like structures, radiating around the central vein, morphological structure appeared normal, with no obvious pathological changes. In livers of METH-treated group, hepatocyte swelling, nuclear fragmentation and disappearance, as well as widespread loss of global cytoplasm, were observed (black arrows in Figure 5B). Compared to METH-treated group, the cellular structure of liver tissue of QR-treated group tended to normalize, and the widespread loss of cytoplasm was alleviated.

Simultaneously, we measured the serum levels of ALT and AST of each group (Figures 5C, D). Compared to control group, the levels of ALT and AST in the METH-treated group significantly increased, indicating severe liver damage in mice (**P < 0.01, ***P < 0.001). Compared to the METH-treated group, ALT and AST levels of QR-treated mice were dramatically decreased with a dose-dependent manner (##P < 0.01, ###P < 0.001). These results collectively demonstrate that QR ameliorates METH-induced acute liver injury in mice.

### 3.5 QR alleviated METH-induced oxidative stress of mice liver tissue

**FIGURE 6:**
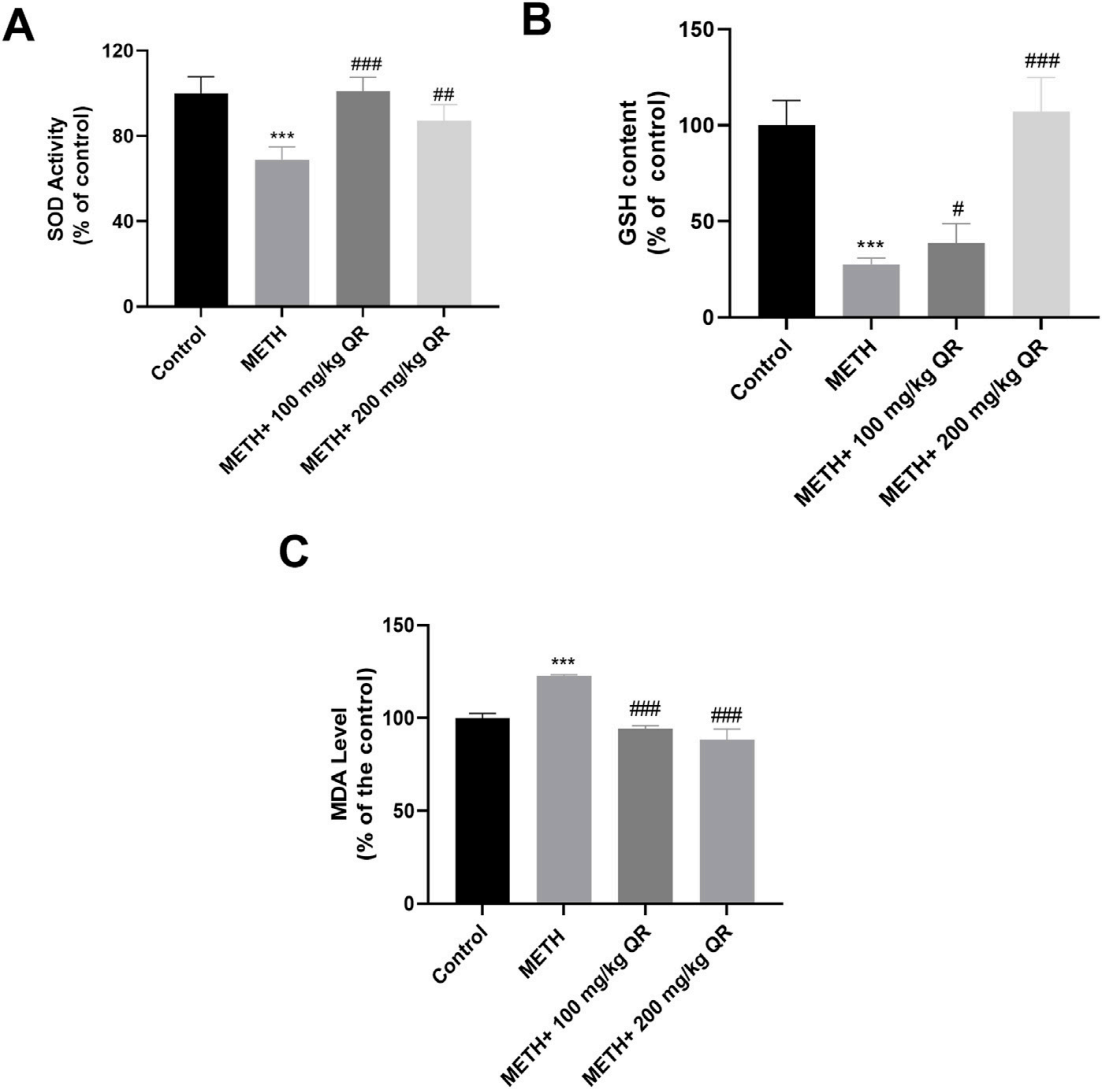
QR alleviates METH-induced hepatic oxidative damage in mice. **(A–C)** Levels of SOD, GSH, and MDA of liver tissues, respectively. Data were shown as the mean ± SD of three independent experiments. ***P < 0.001, compared to Control group. ^#^P < 0.05, ^##^P < 0.01, ^###^P < 0.001, compared to METH-treated group.

We next evaluated the effects of QR on METH-induced oxidative stress of hepatocytes *in vivo*. Separate measurements of SOD, GSH, and MDA levels were made in mouse liver tissue homogenates (Figures 6A–C). Compared to control group, the levels of SOD and GSH of liver in METH-treated group were significantly decreased, while the levels of MDA were increased (***P < 0.001), indicating oxidative damage of liver tissue. However, compared to the METH group, the levels of SOD and GSH of QR-treated group were significantly increased while the levels of MDA were significantly decreased (#P < 0.05, ##P < 0.01, ###P < 0.001). These results indicate that QR alleviates METH-induced liver cell oxidative damage of mice.

### 3.6 QR reduced the METH-induced liver damage via inhibiting mitochondrial injury pathways *in vivo*

**FIGURE 7:**
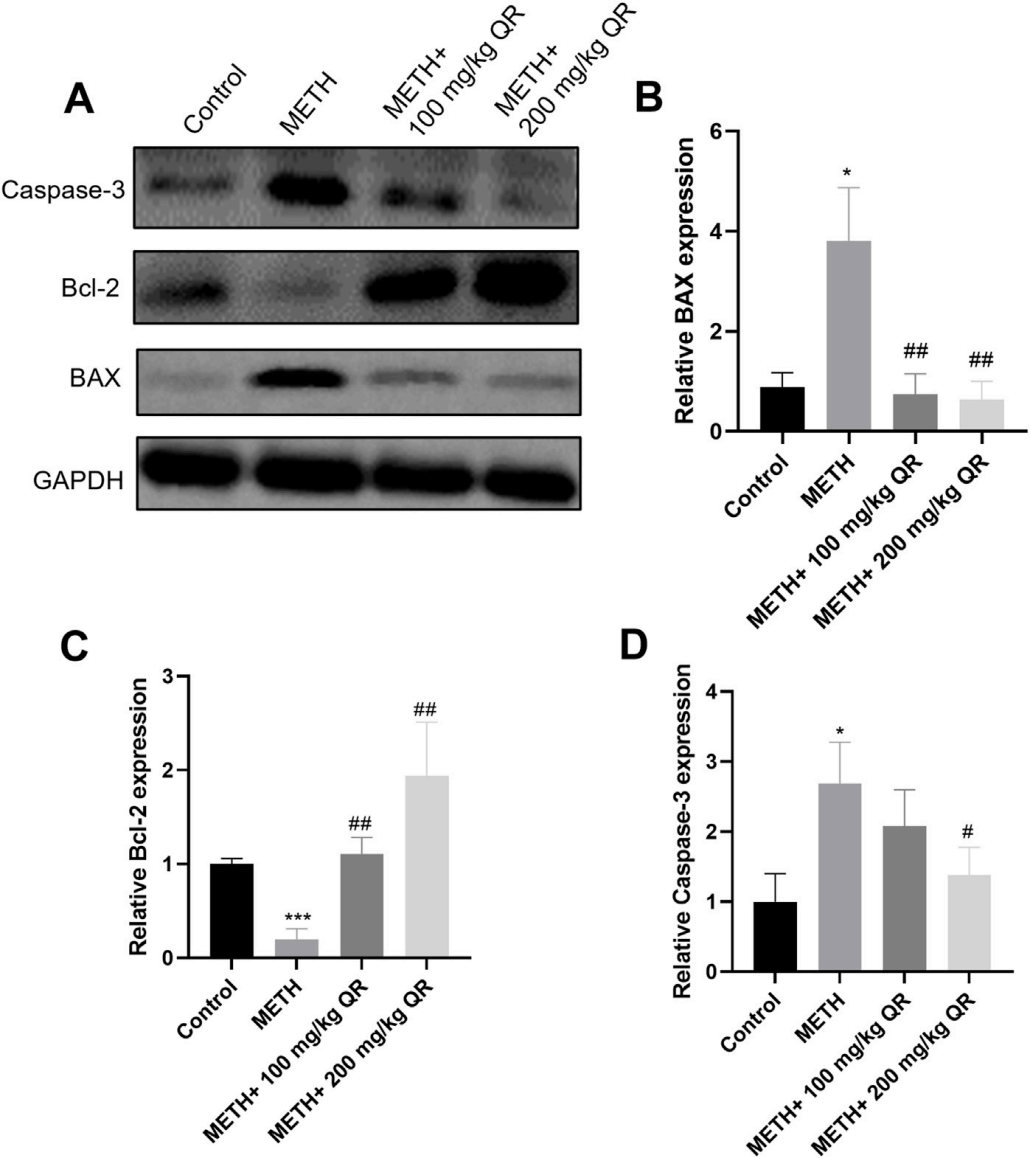
Effects of QR and METH on mitochondrial damage protein expression of liver tissues. **(A)** Western blotting analysis of BAX, Bcl-2, and Caspase-3 levels of mice liver tissues (GADPH was used as internal control). **(B–D)** Statistical plots of gray-scale values of BAX, Bcl-2, and Caspase-3 protein expression (GADPH was used as internal control). Data were shown as the mean ± SD of three independent experiments. *P < 0.05, ***P < 0.001, compared with control group. ^#^P < 0.05, ^##^P < 0.01, compared with METH-treated group.

Furthermore, we investigated the underlying mechanism of how QR alleviates METH-induced acute liver damage of mice. BAX and BCL-2 are mitochondrial proteins whose expression levels change in response to mitochondrial damage, and this alteration in turn leads to the activation of Caspase-3, a protein from the cysteine protease family, thereby accelerating cellular apoptosis (Renault et al., 2013). As shown in Figures 7A–D, it was observed that, compared to control group, METH significantly reduced the levels of the anti-apoptotic protein Bcl-2 and markedly induced the levels of pro-apoptotic proteins BAX and Caspase-3 (*P < 0.05, ***P < 0.001); however, compared to METH-treated group, QR could increase the expression of Bcl-2 protein and inhibit the expression of BAX and Caspase-3 proteins (#P < 0.05, ##P < 0.01). All these findings suggest that QR alleviates METH-induced liver damage and exerts its effects, at least partially, by inhibiting the mitochondrial apoptotic pathway.

## 4 Discussion

The abuse of METH can lead to acute liver injury, with mechanisms of damage largely associated with oxidative stress and mitochondrial impairment (Mashayekhi et al., 2014). AF shows various pharmacological activities, such as anti-inflammatory, anti-depressant, anti-oxidant, and anti-emetic properties (Ebrahimzadeh et al., 2019; Huang et al., 2023; Li and Yang, 2020). The antioxidant capacity of QR, an active component of AF, is significant (Chen et al., 2022). Our study aimed to explore the effects of AF on METH-induced liver injury and the mechanism through which QR alleviates this injury.

Firstly, we observed that AFAE reduced METH-induced hepatocyte viability damage. Further investigation demonstrated that the EA fraction alleviated METH-induced hepatocyte toxicity, highlighting this extraction part as an effective component of AF.

The chemical composition of the ethyl acetate fraction of AF alcohol extract was subsequently analyzed by UPLC-MS/MS. Eight components were identified after comparison with literature and databases. Among the eight major components, QR showed highest part of the total area of the EA fraction.

QR possesses various pharmacological activities including anti-oxidant, anti-viral, anti-tumor, anti-inflammatory, and anti-analgesic effects (Sheu et al., 2009; Li et al., 2017; Dewi et al., 2019; Tang et al., 2019; Chen et al., 2022; ). We found that, METH triggers apoptosis in liver cells and induces cells arrest in the G0/G1 phase, while QR significantly alleviated these damages. Numerous studies have shown that METH significantly induce oxidation of liver (Bowyer and Hanig, 2014). Mashayekhi et al. (2014) found that METH may induce an increase in ROS levels, leading to mitochondrial damage and accelerated cell apoptosis. Our data showed that QR alleviates METH-induced oxidative damage. This effect was also confirmed in *in vivo* animal experiments.

Mitochondria are essential for initiating apoptosis, and ROS regulate mitochondrial homeostasis under normal conditions. When ROS production increases, it may induce a decrease in mitochondrial membrane potential and further exacerbate apoptosis (Bock and Tait, 2020). We found that QR significantly inhibits the intracellular ROS levels induced by METH and decreases mitochondrial membrane potential.

METH-mediated ROS generation may associated with intracellular calcium homeostasis disruption. Previous studies showed that METH disrupts the calcium homeostasis within hepatocytes (Lee et al., 2012). This disruption leads to an abnormal influx of calcium ions into mitochondria (Smith et al., 2014). Excessive intramitochondrial calcium activates calcium-dependent proteases and phospholipases (Garcia et al., 2010). These activated enzymes initiate a series of events that culminate in the damage of the mitochondrial membrane (Garcia et al., 2010; Lee et al., 2012). Specifically, mitochondrial cristae disorganization, a decrease in mitochondrial membrane potential, and an increase in ROS production occur (Lee et al., 2012). The combined effect of these changes may ultimately lead to mitochondrial damage and subsequent cell apoptosis.

QR may regulate ROS levels by activating the nuclear factor erythroid 2 - related factor 2 (Nrf2) pathway to alleviate METH-induced oxidative injury of liver cells. Previous studies have shown that QR could protect against oxidative stress in epithelial cells, brain tissues, and lung tissues (Sun et al., 2020; Jia et al., 2021; Cheng et al., 2024; Yousefi Zardak et al., 2024). Once activated, Nrf2 translocates to the nucleus and binds to the antioxidant response element (ARE), this binding event subsequently promotes the transcription of genes encoding antioxidant enzymes, such as SOD and glutathione peroxidase (GPx) (Yousefi Zardak et al., 2024). The upregulation of these antioxidant enzymes may effectively scavenge the excessive ROS generated due to METH exposure, which in turn alleviates oxidative stress and subsequent mitochondrial damage of liver cells., thereby exerting a protective effect against METH-induced hepatotoxicity.

**FIGURE 8:**
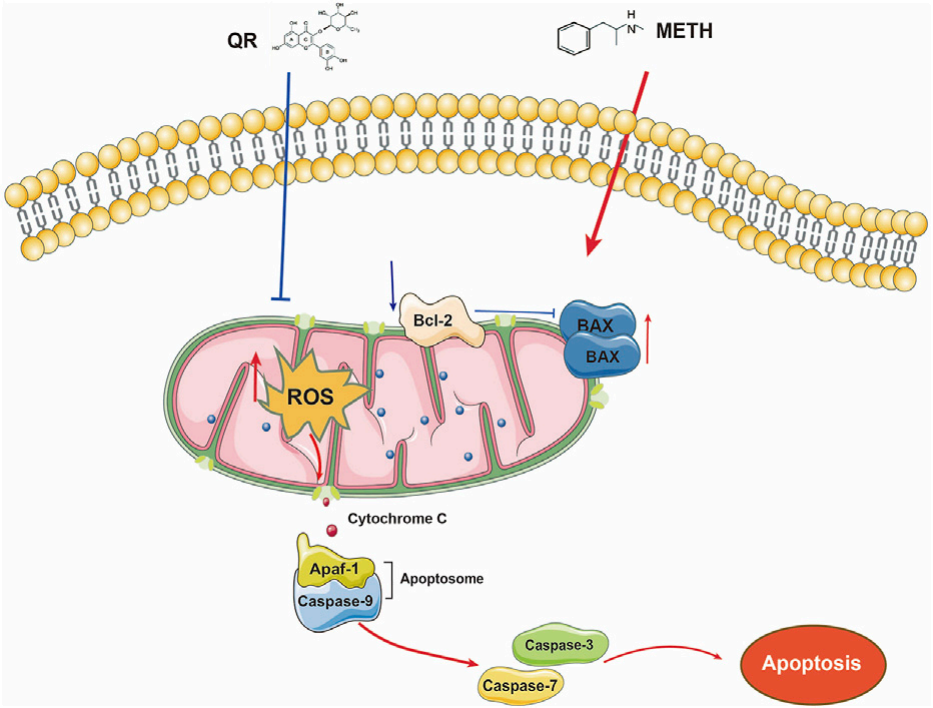
The mechanism of how QR reduces METH-induced hepatotoxicity. METH damages the mitochondria of hepatocytes, triggering the release of ROS. Elevated ROS levels cause a decrease in mitochondrial membrane potential, disrupt the balance between pro-apoptotic and anti-apoptotic proteins, promote the formation of apoptotic bodies, and activate Caspase-3 protein, ultimately accelerating apoptosis. QR, however, acts to counteract these detrimental effects by inhibiting the release of ROS, thereby preventing mitochondrial dysfunction and apoptosis. By mitigating ROS-induced damage and preserving mitochondrial integrity, QR effectively suppresses METH-induced hepatotoxicity.

Upon activation, BAX/BCL-2 proteins trigger the release of cytochrome C from mitochondria, inducing the formation of apoptotic bodies, activating downstream effector molecules such as caspase-3/7, initiating a protease cascade reaction, and leading to cell disintegration (Taylor et al., 2008). Our results indicate that METH significantly increases the expression of pro-apoptotic proteins BAX and caspase-3 while decreasing the expression of anti-apoptotic protein Bcl-2. Following QR treatment, this trend is reversed, demonstrating its inhibitory effect on METH-induced liver damage. Overall, our study found that QR mitigates METH-induced liver damage through the mitochondria-mediated intrinsic apoptotic pathway (Figure 8).

## 5 Conclusion

Our study focused on investigating the effects of QR, an active constituent of AF, on METH-induced acute liver injury. *In vitro* and *in vivo* results demonstrated that QR significantly ameliorated METH-induced hepatocyte damage and liver dysfunction. Additionally, we observed that QR inhibited METH-induced liver cell toxicity by modulating BAX/CASP3 signaling pathway. Our study provides a theoretical foundation for the development of therapeutic agents targeting METH-induced liver injury and explores the therapeutic potential of natural herbal medicines in alleviating liver damage. However, a comprehensive evaluation of QR’s benefits requires further investigation to assess additional biomarkers and elucidate the detailed mechanisms by which QR alleviates METH-induced liver injury.

## Data Availability

The original contributions presented in the study are included in the article/Supplementary Material, further inquiries can be directed to the corresponding authors.
